# Perspectives of CRISPR/Cas-mediated *cis*-engineering in horticulture: unlocking the neglected potential for crop improvement

**DOI:** 10.1038/s41438-020-0258-8

**Published:** 2020-03-15

**Authors:** Qiang Li, Manoj Sapkota, Esther van der Knaap

**Affiliations:** 1 College of Horticultural Science and Engineering, Shandong Agricultural University, Tai’an, China; 2 Center for Applied Genetic Technologies, University of Georgia, Athens, GA USA; 3 Institute for Plant Breeding, Genetics and Genomics, University of Georgia, Athens, GA USA; 4 Department of Horticulture, University of Georgia, Athens, GA USA

**Keywords:** Molecular engineering in plants, Plant breeding, Molecular engineering in plants, Plant breeding

## Abstract

Directed breeding of horticultural crops is essential for increasing yield, nutritional content, and consumer-valued characteristics such as shape and color of the produce. However, limited genetic diversity restricts the amount of crop improvement that can be achieved through conventional breeding approaches. Natural genetic changes in *cis*-regulatory regions of genes play important roles in shaping phenotypic diversity by altering their expression. Utilization of CRISPR/Cas editing in crop species can accelerate crop improvement through the introduction of genetic variation in a targeted manner. The advent of CRISPR/Cas-mediated *cis*-regulatory region engineering (*cis*-engineering) provides a more refined method for modulating gene expression and creating phenotypic diversity to benefit crop improvement. Here, we focus on the current applications of CRISPR/Cas-mediated *cis*-engineering in horticultural crops. We describe strategies and limitations for its use in crop improvement, including de novo *cis*-regulatory element (CRE) discovery, precise genome editing, and transgene-free genome editing. In addition, we discuss the challenges and prospects regarding current technologies and achievements. CRISPR/Cas-mediated *cis*-engineering is a critical tool for generating horticultural crops that are better able to adapt to climate change and providing food for an increasing world population.

## Introduction

Horticultural crops comprise vegetables, fruits, and ornamental flowers as well as aromatic and medicinal plants, thereby providing essential resources to society. For example, the availability and consumption of a wide variety of vegetables and fruits allow us to meet our daily dietary needs. Moreover, we enlighten our days with the abundance of floriculture products for aesthetic uses and visual enjoyment. Collectively, horticultural crops make essential contributions to humankind while also providing the economic engines that drive the success of societies all over the world^1^.

Despite their collective importance, the improvement of many horticultural crops has lagged behind most agronomic crops, such as rice, corn, and soybean. Yet, improvement of horticultural crops for traits such as resistance to biotic and abiotic stresses, yield, and health-related nutrients would benefit the entire sector. Genetic diversity is a critical source for crop improvement. However, this diversity is often limiting, especially for certain species^2^. The limited genetic diversity could result in significant obstacles for further improvement by conventional breeding approaches. Research in several crops has demonstrated that much of the genetic changes underlying traits of economic importance reside in the *cis*-regulatory regions of genes^3,4^. These changes appear to have been selected during domestication, resulting in desirable traits caused by altered gene expression^3,5^. The CRISPR/Cas-based platform offers a powerful tool by engineering *cis*-regulatory regions (*cis*-engineering) to introduce genetic diversity that could potentially accelerate crop improvement^6–10^. Despite the importance of regulatory changes in genes, the application of CRISPR/Cas-mediated *cis*-engineering has only been explored sporadically. The genome sequence for at least 181 horticultural species is available^11^ and genome editing has been used to generate primarily knockout mutations in at least 25 of them^12–17^. These achievements demonstrate the feasibility of applying CRISPR/Cas-mediated *cis*-engineering to expand the phenotypic diversity of many horticultural crops.

## Natural variation in *cis*-regulatory regions resulting from the domestication of horticultural crops

*Cis*-regulatory regions are non-coding DNA sequences that control the transcription of genes^18^. These *cis*-regulatory sequences consist of combinations of CREs that affect gene expression level often in a spatiotemporal manner^9,18,19^. Single-nucleotide polymorphisms (SNPs), insertions, deletions, inversions, and epigenetic variations are the most common natural variation in *cis*-regulatory regions that are associated with domestication. Some examples from horticultural crops are discussed below.

### Single-nucleotide polymorphisms

Genomic studies in horticultural crops have generated insights into the role of SNP in shaping phenotypic diversity among individuals^20^. During tomato (*Solanum lycopersicum*) domestication, selection frequently occurred for fruit size and shape, traits that show extensive variation and large increases over that of the wild relatives^4^. Increases in fruit weight are thought to be controlled by SNPs in the promoter of *FW2.2* (*SlCNR*) and *FW3.2* (*SlKLUH*)^4,21,22^. The *lc* allele contains two SNPs in a 15-bp repressor element downstream of tomato *WUSCHEL* (*SlWUS*). The SNPs are proposed to prevent the binding of the MADS-box transcription factor AGAMOUS, which is required to recruit the repressive Polycomb proteins to shut down *SlWUS* expression, thereby ultimately resulting in larger fruits^4,23,24^. In another example in tomato, two SNPs in the promoter of *Slcyc-B* are highly associated with high β-carotene content^25^.

In citrus (*Citrus reticulata*), a recent report found an SNP in a miniature inverted-repeat transposable element (MITE) in the promoter of *carotenoid cleavage dioxygenase 4b* (*CCD4*) to be sufficient to increase the expression of this gene, resulting in red coloration of fruit peel^26^. In pepper (*Capsicum chinense*), an SNP in the promoter of *MYB31* is associated with a hyperfunctional W-box, leading to stronger binding of WRKY9. This stronger binding is associated with enhanced expression of *MYB31*, resulting in extremely pungent peppers^27^.

### Insertions

Insertions are sources of genetic diversity that can alter gene expression by introducing new or disrupting existing CREs. Especially transposable elements (TEs) play important roles in creating genomic variation by altering gene regulation^28,29^. TE-induced variations in *cis*-regulatory region are also important in the shaping of domestication-related phenotypes in many horticultural crops. One example is the tomato fruit shape gene *SUN*. The transposition event at the *sun* locus mediated by the *Rider* retrotransposon placed a copy of *SUN* in addition to *Rider* itself in the intron of *DEFL1*. The ancestral copy of *SUN* on chromosome 10 is lowly expressed, but its derived copy on chromosome 7, where the *sun* locus maps, is highly expressed^30^. The high expression of *SUN* on chromosome 7 is thought to be from the promoter of *DEFL1* that would now serve as an enhancer of *SUN*, leading to the elongated tomato fruit^31^. Another *Rider* insertion in the first intron of *SEPALLATA4* (*SEP4*) leads to a jointless pedicel, reduced fruit dropping, which facilitates mechanical harvesting^32^. In grape (*Vitis vinifera*), the insertion of the *Gret1* (Grapevine Retrotransposon 1) in the *VvMYBA1* promoter leads to its inactivation, resulting in a white berry phenotype^33^. In blood oranges (*Citrus sinensis*), the insertion of a *Copia*-like retrotransposon controls the expression of *Ruby* and the cold dependency of anthocyanin production in the fruit^34^. In cauliflower (*Brassica oleracea* var *botrytis*), a 695-bp *Harbinger* DNA transposon insertion in the *MYB2* promoter increases the expression of this gene, resulting in a purple phenotype^35^. Additionally, the differentiation of winter and spring genotypes in rapeseed (*Brassica napus* L.) primarily arose from a MITE transposon insertion in the upstream region of *BnFLC.A10*^36^.

Other examples of insertions that are possibly associated with TE activity are found as well. In tomato, *ej2*^*w*^ (*enhancer-of-jointless 2*) is a weak loss-of-function allele, which was selected during tomato domestication and caused by a 564-bp insertion in the fifth intron of *EJ2*. The mutation results in unbranched inflorescences with exceptionally large sepals^32^. An 8-bp insertion in the promoter of *SlbHLH59* significantly increased its expression in accessions producing high ascorbic acid levels^37^. In apple (*Malus* × *domestica*), multiple repeats of a 23-bp motif in the promoter of *MYB10* generate an autoregulatory locus, which is sufficient to account for increased expression and ectopic accumulation of anthocyanins in red-fleshed apples^38^. Another example from apple is that a 36-bp insertion in *MdSAUR37* promoter contributed to high fruit malate content^39^. In cucumber (*Cucumis sativus* L.), a 10-bp fragment was replaced by an 812-bp fragment in the promoter of *CsHDZIV11*/*CsGL3* at the *few spines 1* (*fs1*) locus, giving rise to higher expression of *CsGL3* and fewer fruit spines^40^.

### Deletions and inversions

Deletions are common genetic changes that provide a wealth of domesticated related phenotypic diversity. One remarkable example is a 31-kb deletion upstream of tomato *OVATE Family Protein 20* (*SlOFP20*). The deletion is associated with reduced expression of *SlOFP20* and contributes to natural fruit shape variation in the tomato germplasm^41^. A 3-bp deletion in the promoter of tomato *Al-ACTIVATED MALATE TRANSPORTER9* (*Sl-ALMT9*) was selected during tomato domestication. The deletion disrupts the repression of *Sl-ALMT9* by Sl-WRKY42. This results in increased *Sl-ALMT9* gene expression levels, thereby conferring high fruit malate contents and aluminum tolerance in tomato^42^. Flowering time is an important trait for cucumber domestication. A 39.9-kb deletion and a 16.2-kb deletion located 16.5-kb upstream of cucumber *FLOWERING LOCUS T* (*CsFT*) are both associated with higher *CsFT* expression levels and earlier flowering^43^. The *CsFT* locus was selected during cucumber domestication and has been important in its adaptation to higher latitudes for cultivation^43^. Therefore, deletions can confer desirable traits through either decreased gene expression by removing enhancers and binding sites of activators or increased gene expression by removing repressors and binding sites of repressors.

Genomic inversions also play a role in plant domestication as they could have widespread *cis*-regulatory effects^44^. One of the remarkable examples of variation in locule number is controlled by a nearly 300-kb inversion of the *fasciated* (*fas*) locus in tomato. The *fas* locus is characterized by disruption of the promoter region of tomato *CLAVATA3* (*SlCLV3*), leading to downregulation of the gene and larger fruit with increased number of locules^24,45^.

### Epigenetic variations

Natural epigenetic variations contribute to heritable phenotypic diversity that is not caused by modification in the DNA sequence^46–49^. One of the best examples of an epiallelic variant that impacts an important agronomical trait is the *Colorless Non-Ripening* (*Cnr*) allele in tomato. The epiallele of *LeSPL-CNR* is responsible for colorless fruits with a substantial loss of cell-to-cell adhesion^50^. In *Cnr* mutants, hyper-methylation was found along a 286-bp CRE located ~2.4-kb upstream from the first ATG of *LeSPL-CNR*. This change in methylation status likely explains the reduced expression level of *LeSPL-CNR* and the ripening defects in *Cnr* fruits^50^. Another epigenetic mutation was found in the promoter of the tomato *SlTAB2* gene. The mutation controls pigment production in tomato leaves that are affected by DNA methylation level in the promoter of the gene^51^. *Vitamin E* 3 (*VTE3*) is another naturally occurring epiallele controlling vitamin E accumulation in tomato fruits^52^. The *VTE3* expression in fruits is regulated by DNA methylation in the promoter region of the gene^52^. Additional examples include the control of anthocyanin accumulation in apple and pear (*Pyrus communis*) fruit skin^53–55^ and sex determination in melon (*Cucumis melo*)^56^. There is also increasing evidence that promoter DNA methylation plays an important role in regulating tomato fruit ripening^57,58^. Notably, the tomato *DML2* is critical for tomato fruit ripening by mediating DNA hypomethylation in promoters of hundreds of genes during development^58^.

Taken together, these studies highlight the importance of genetic and epigenetic divergence in *cis*-regulatory regions, including the upstream regions, introns, and downstream regions of genes. Therefore, natural genetic variants, epialleles, and functional CREs in *cis*-regulatory regions are excellent genome editing targets to create novel variants for the improvement of horticultural crops.

## Recent progress in CRISPR/Cas-mediated *cis*-engineering in plants

So far, the most frequent application of CRISPR/Cas has been to target coding sequences with the goals to create null alleles^59–62^. Although this application greatly facilitates heritable alleles for reverse genetics studies, selection of loss-of-function mutations in coding regions may result in pleiotropic or deleterious effects^45,63,64^. Compared to coding sequences, modulating gene expression by *cis*-engineering is more likely to benefit crop improvement with less detrimental pleiotropic effects^3,7,9,10,59,64^.

**Fig. 1:**
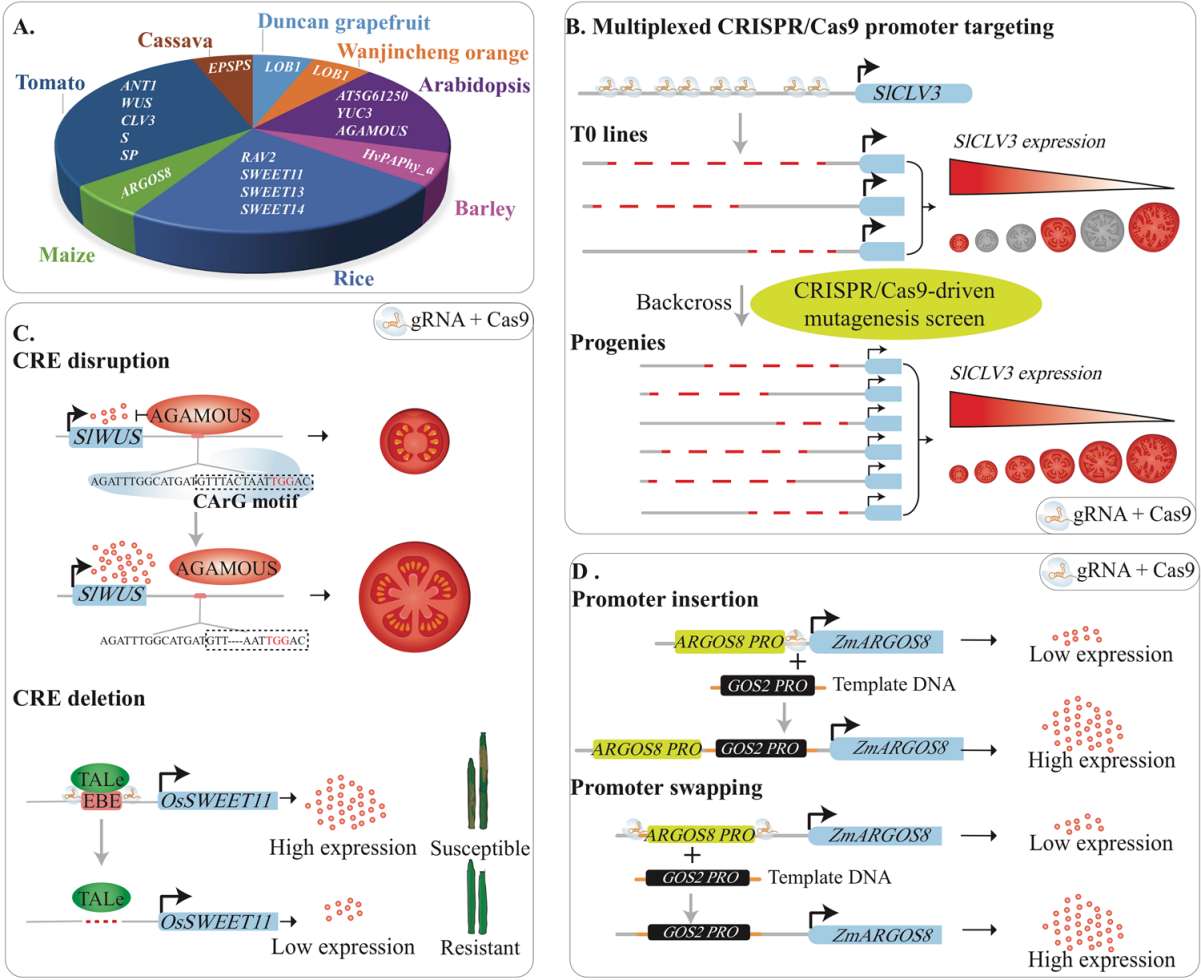
Current applications of CRISPR/Cas-mediated *cis*-engineering. **a** Summarization of current applications of CRISPR/Cas-mediated *cis*-engineering in plants. **b** A continuum of phenotypic variation can be achieved by multiplexed CRISPR/Cas9 promoter targeting and sensitized genetic screen. **c** Disruption of CREs with genome editing can generate gain-of-function and reduced or loss-of-function alleles. **d** HDR-mediated promoter insertion/swapping conferring higher gene expression resulting in desirable traits. *LOB1*, *LATERAL ORGAN BOUNDARIES 1*; *YUC3*, *YUCCA3*; *ARGOS8*, *Auxin-Regulated Gene Involved in Organ Size 8*; *ANT1*, *Anthocyanin 1*; *WUS*, *WUSCHEL*; *CLV3*, *CLAVATA3*; *S*, *COMPOUND INFLORESCENCE; SP*, *SELF PRUNING*; *SWEET*, *SUGARS WILL EVENTUALLY BE EXPORTED TRANSPORTERS*; *EPSPS*, *5-enolpyruvylshikimate-3-phosphate synthase*; TALe, Transcription-activator-like effector; EBE, Effector-binding element; CRE, *Cis*-regulatory element; *PRO*, promoter.

To date, at least 15 articles described successful CRISPR/Cas-mediated *cis*-engineering via genome editing for 17 genes in eight plants species, including eight genes in four horticultural crops (Fig. 1a). In addition, CRISPR/Cas-mediated *cis*-engineering has also been achieved to edit the epigenome. However, only a handful of cases have been described in *Arabidopsis* that show epigenome editing by altering DNA methylation^65,66^ and histone acetylation^67^. Because of the few examples in epigenome editing, the following sections will only describe the applications of *cis*-engineering of DNA.

### Promoter disruption

In tomato, a multiplexed CRISPR/Cas9 promoter targeting approach was used to edit the promoters of genes that control fruit size, inflorescence branching, and plant architecture^7^. Importantly, this approach did neither exploit nor require prior knowledge regarding the structure of promoters and other regulatory sequences. Therefore, the multiplexed CRISPR/Cas9 promoter targeting approach is generally applicable for diverse genes and traits in many crops. Notably, a CRISPR/Cas9-driven sensitized genetic screen approach can recover a collection of *cis*-regulatory alleles with a continuum of phenotypic effects^7^ (Fig. 1b), providing an avenue for expanding genetic diversity in crops.

### CRE disruption/deletion

Functional CREs in *cis*-regulatory regions are obvious targets for expanding genetic diversity. However, only a handful of cases have been reported in plants, in which the CRE disruption/deletion was successfully applied to regulate target gene expression.

The rice *RAV2* gene is transcriptionally regulated by high salinity. CRISPR/Cas-mediated *cis*-engineering was used to target the GT-1 element in the promoter of *OsRAV2* and the results strongly indicate that the GT-1 element controls the salt response of this gene^68^. In barley (*Hordeum vulgare*), the promoter of *HvPAPhy_a* was targeted for three CREs, namely GCN4, Skn1, and RY^69^. The lines with mutations in the targeted region show a significant reduction in phytase activity, indicating the importance of these CREs for the expression of the gene. Similarly, the deletion of a 149-bp regulatory fragment containing a transcription-activator-like effector (TALe)-Binding Element (EBE) in the promoter of *SUGARS WILL EVENTUALLY BE EXPORTED TRANSPORTERS 11* (*SWEET11*) improved rice disease resistance without affecting rice fertility^64^ (Fig. 1c). This result is advantageous compared to the knockout mutant of *OsSWEET11* that showed a sterile phenotype, which is obviously undesirable in crop improvement. Recently, simultaneously editing of EBEs in the promoters of *SWEET* genes resulted in rice lines with broad-spectrum bacterial blight resistance^70,71^. Three recent studies in Duncan grapefruit (*Citrus paradisi* Macf.) and Wanjincheng orange (*Citrus sinensis* Osbeck) reported that canker‐resistant plants were created through CRISPR/Cas editing of the PthA4 effector binding CREs in the promoter of *LATERAL ORGAN BOUNDARIES 1* (*LOB1*)^72–74^.

The CRISPR/Cas-mediated *cis*-engineering was also utilized to modify known CREs in introns and downstream of genes. The disruption of the CArG element, including the two causative SNPs downstream of *SlWUS*, is one of the remarkable examples recreating gain-of-function alleles^7,75^ (Fig. 1c). In *Arabidopsis*, a CTCTGYTY motif in the intron of *YUCCA3* (*YUC3*) was identified by chromatin immunoprecipitation-sequencing (CHIP-seq) and is crucial for recruiting RELATIVE OF EARLY FLOWERING 6 (REF6) to its target loci^76–78^. The deletion of four repeats of this motif leads to diminished binding of REF6 at the mutant loci. In addition, a 450-bp CRE in the second intron of *Arabidopsis* *AGAMOUS* (*AG*) was deleted by CRISPR/Cas9 and verified as the activator of *AG* gene expression. The deletion of this CRE resulted in early flowering because of a 40% decrease in its expression^79^.

### Promoter insertion/swapping

Promoter insertion and swapping can be achieved by homology-directed repair (HDR) with potentially great importance to crop improvement (Fig. 1d). However, HDR has been challenging due to its low efficiency in higher plants^60,80^. So far, only three cases have been reported, in which the promoters were accurately inserted or swapped by CRISPR/Cas9-mediated HDR^81–83^. A 35S promoter was inserted upstream of *anthocyanin 1* (*ANT1*), resulting in enhanced anthocyanin accumulation and intensely purple tomato tissues^81^. In maize, the HDR pathway was used to insert as well as swap the native *GOS2* promoter in the 5′-untranslated region of *ARGOS8* (Fig. 1d). The edited plants showed increased expression of *ARGOS8* and higher grain yield under drought stress conditions in field trials^82^. Additionally, glyphosate-tolerant cassava (*Manihot esculenta*) was generated by a promoter swap of the *5-enolpyruvylshikimate-3-phosphate synthase* (*EPSPS*) gene^83^.

These encouraging achievements show the potential for using CRISPR/Cas-mediated *cis*-engineering to improve crop yield, quality, and stress resistance.

## Strategies for application of CRISPR/Cas-mediated *cis*-engineering in horticultural crops improvement

### De novo CRE discovery

Prior knowledge of CREs in *cis*-regulatory region is helpful to apply *cis*-engineering in crop improvement. Many previously described CREs, especially transcription factor-binding sites (TFBSs), in plant promoters can be identified by submitting sequences to various databases, including TRANSFAC^84^, PLACE^85^, PlantCARE^86^, JASPAR Core PLANTAE^87^, PlantTFDB^88^, and Plant Regulomics^89^. After the TFBSs have been predicted, the regions can be validated by either in vitro methods based on DNA–protein interaction, such as DNA electrophoretic mobility shift assay, DNA pull-down and yeast one-hybrid assays, or in vivo CHIP-based methods, for example, CHIP with DNA microarray (CHIP-chip) and CHIP-seq.

However, the vast majority of CREs are unknown or poorly characterized, highlighting the pressing need for de novo CRE discovery. The availability of genomic and transcriptomic data for many horticultural crops allows the identification of novel CREs using bioinformatics-based and experimental approaches^11,90,91^. The de novo CRE discovery is based on sequence conservation that exists among groups of genes that are co-expressed as well as gene families within a single genome, and among orthologs of multiple species^91–93^.

Genes that show similar expression patterns or are in the same gene family are likely to be tightly co-regulated and/or functionally related. Therefore, clustering co-expressing genes and identification of gene families are helpful to explore conserved CREs and uncover their functions for transcriptional regulation. The shared CREs can be identified by the well-established methods such as multiple EM for motif elicitation (MEME)^94,95^ and eXhaustive evaluation of matriX motifs (XX motif)^96,97^. An ensemble strategy was used for de novo soybean cyst nematode-inducible motif discovery in the upstream regulatory sequences of 18 co-expressed genes^98^. Another strategy to identify conserved CREs is by comparing promoter sequences of orthologous genes from different species. Phylogenetic footprinting and variations of the technique are designed for the CRE discovery approach^99–103^. mVISTA is a commonly used tool for comparative analysis of genomic sequences^104^. The comparison of the *CLV3* promoters in tomato with three other *Solanaceae* species, *S. pennellii*, potato (*S. tuberosum*), and pepper (*C. annuum*) was performed using mVISTA. This resulted in the identification of three putative CREs between tomato and pepper, and four CREs between tomato and potato^7^. Complementary to bioinformatics-based approaches are experimental approaches, for example, deconstructive and reconstructive approaches, by which numerous inducible and tissue-specific CREs are characterized^90,105^.

### Choice of appropriate approach for CRISPR/Cas-mediated *cis*-engineering

CRISPR/Cas-based technologies offer multiple strategies to engineer *cis*-regulatory regions according to the prior knowledge of the target region or given purpose. If no prior knowledge of the target region exists, multiplexed CRISPR/Cas promoter targeting approach can be applied to putative “negative regulators” of the desirable traits by creating a collection of reduced-function alleles (Fig. 1b). In addition, a well-defined promoter can be exchanged with the promoter of the gene of interest to increase expression level or change temporal/spatial expression pattern of the gene (Fig. 1d). For a given CRE in a target region of interest, the CRE can be disrupted or deleted on the basis of the random indel mutations introduced by non-homologous end joining (NHEJ) repair pathway^7,64,69,72–75,78,79^ (Fig. 1c).

**Fig. 2:**
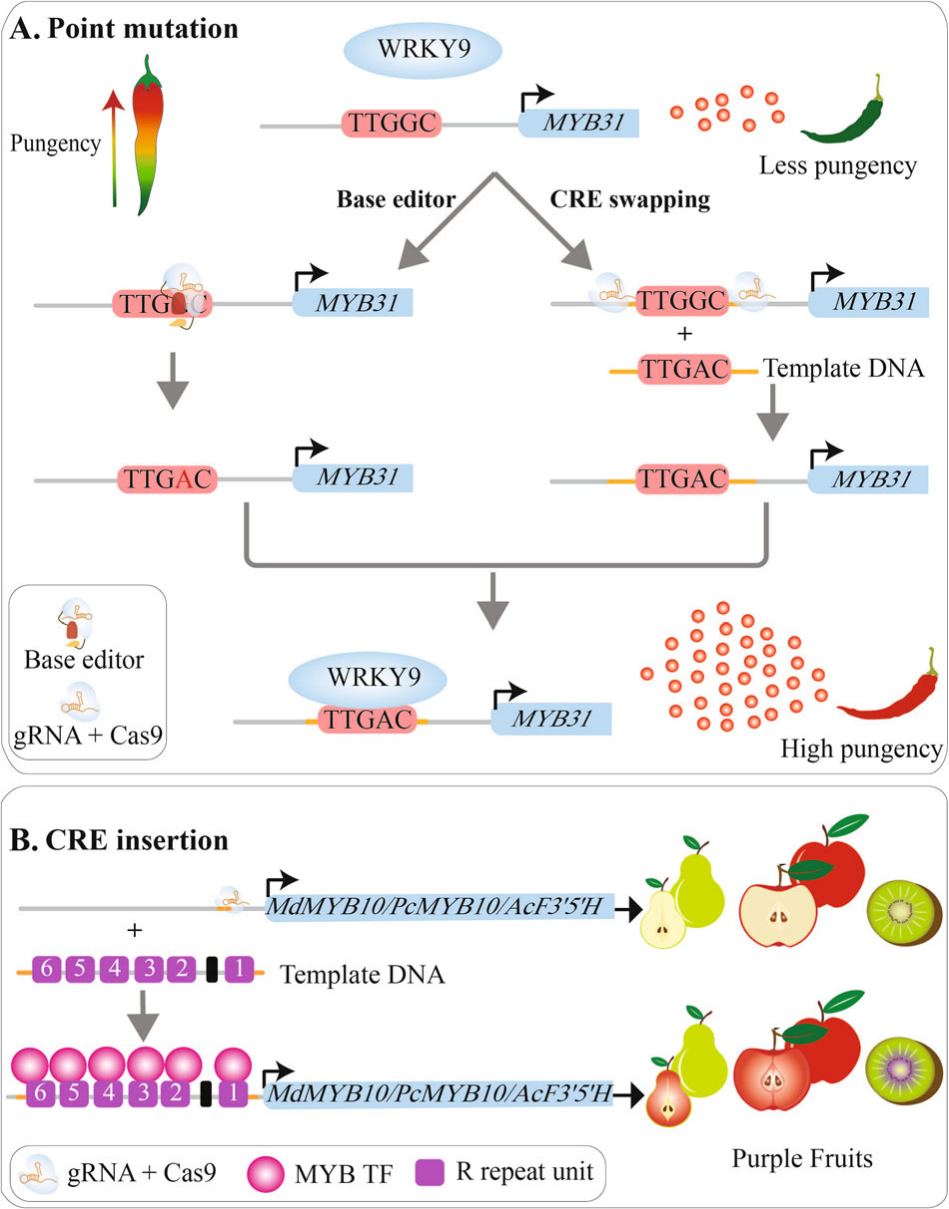
Examples of the potential applications of CRISPR/Cas-mediated *cis*-engineering in horticultural crops. **a** CRISPR/Cas-mediated point mutations can be achieved by base editor or HDR-mediated CRE swapping. In some *Capsicum* species, a mutated W-box in the *MYB31* promoter is not recognized by the activator WRKY9. Base editor and CRE swapping can change the motif TTGGC to W-box (TTGAC), which can be bound by WRKY9, resulting in increased expression of *MYB31* and higher pungency level. **b** The R6 motif insertion mediated by HDR confers *trans*-regulation by flavonoid-related MYBs, which can bind the R6-containing promoters of the genes encoding enzymes of the anthocyanin biosynthetic pathway, resulting in enhanced expression of these genes and higher anthocyanin levels. CRE, *cis*-regulatory element; F3′5′H, flavonoid 3′5′-hydroxylase.

CRISPR/Cas-mediated point mutations and CRE swaps are also important approaches to manipulate gene expression (Fig. 2). Apart from the above-mentioned SNPs that underlied the domestication of crops, numerous studies also documented that single-nucleotide alterations in regulatory sequences can be sufficient to produce substantial effects on gene expression^106–108^. For example, in soybean, nucleotide mutations in the core and flanking sequences of G-box element lead to both increases and decreases in gene expression in both native and synthetic promoters^109^. In apple, the presence of R6 motif, a binding site of *MdMYB10*, in the promoter of *MdMYB10* results in auto-activation of the gene and elevated anthocyanins^38^. The synthetic promoters of pear *MYB10* and *Arabidopsis MYB75* harboring the R6 motif significantly increase the expression of these genes, leading to elevated anthocyanin levels in transgenic plants of pear and *Arabidopsis*^110^. Moreover, the insertion of the R6 motif into the promoter of the gene encoding an anthocyanin biosynthetic enzyme flavonoid 3′5′ -hydroxylase (F3′5′H) and a vitamin C biosynthesis gene *GDP-L-Galactose Phosphorylase* (*GGP*) of kiwifruit (*Actinidia eriantha*) altered the anthocyanin profile and increased vitamin C content in a *MYB10*-dependent manner, respectively^110^. Therefore, the R6 motif can be harnessed to generate new diversity in many horticultural species to increase anthocyanin content (Fig. 2b).

### Transgene-free genome editing

Transgene-free genome editing is the preferred choice for the application of *cis*-engineering for crop improvement and commercialization of genome-edited crops. Genome editing with stable expression of CRISPR/Cas DNA involves the integration of the construct into the host genome, raising concerns about undesirable off-target changes and biosecurity^60,111^. Genetic segregation by selfing or crossing can be used to obtain transgene-free edited plants. Recently, several strategies have been developed to accelerate the removal of transgene components while retaining the desired mutations. These strategies include the integration of fluorescent markers^112,113^, herbicide-dependent isolation system^114^, and the programmed self-elimination system^115^.

An alternative approach for creating transgene-free edited plants is transient expression of CRISPR/Cas DNA as have been reported in many crops, including wheat^116,117^, barley^118^, tetraploid potato^119,120^, tomato^121^, and cotton^122^. Compared to stable transformation of CRISPR/Cas DNA, transient expression is especially useful in certain horticultural crops that are vegetatively propagated, self-incompatible, polyploid, and/or have long juvenile stages^123^.

Given that traditional breeding, including chemically and physically induced mutagenesis, and DNA-based genome editing may introduce off-target mutations, editing in a DNA-free manner via preassembled Cas9 protein-guide RNA (gRNA) ribonucleoproteins (RNPs) is an increasingly popular approach due to higher specificity, and low off-target mutations further alleviating public concerns^124–127^. RNPs have been adopted in the transformation of protoplasts in some horticultural crops, such as lettuce (*Lactuca sativa* L.)^128^, *petunia*^129^, apple and grape^130^, and potato^131^. However, regeneration of mature plants from the edited protoplasts is still challenging for most of the horticultural crops.

Currently available transgene-free genome editing approaches are primarily conducted through traditional transformation methods that require tissue culture, which is a labor-intensive process. Therefore, tissue culture-free methods are highly desirable and necessary for transgene-free genome editing. *In planta* transformation takes advantage of natural biological processes, which makes it a valuable alternative to in vitro tissue culture and regeneration^132,133^. Various plant cells or tissues can be the ideal transformation targets such as germline or meristematic cells^116,134,135^ and dormant buds^136^. Recently, *in planta* particle bombardment has been used to deliver CRISPR/Cas9 DNA into shoot apical meristems, resulting in transgene-free homozygous mutated wheat plants^134^. Moreover, recent efforts have been made to deliver RNPs into embryo cells in maize^135^ and wheat^116^ by particle bombardment and into zygotes by polyethylene glycol-Ca^2+^-mediated transfection in rice^127^.

## Challenges and prospects

### Genome complexity of horticultural crops

The genome sizes of horticultural crops are diverse, ranging from ~200-Mb in some crops, for example, peach (*Prunus persica*), to over 30-Gb in garlic (*Allium sativum*) and onion (*Allium cepa*)^11^. Many horticultural crops underwent polyploidy, posing extra challenges for genome editing using CRISPR/Cas technologies. Genome editing of polyploid crops requires increased efficiency to edit multiple alleles simultaneously. Even so, CRISPR/Cas technologies have been successfully applied in many polyploid crops due to continuous improvements, including highly active Cas nuclease, multiplex genome editing, and efficient expression systems^63,137,138^. In case of octoploid and highly heterozygous cultivated strawberry (*Fragaria* × *ananassa* cv. Camarosa), all five alleles of *FaTM6* were successfully edited using CRISPR/Cas9-mediated dual single-guide (sg) RNA approach^139^. Although the genome of *Fragaria* × *ananassa* is not yet available, the diploid wild strawberry *F. vesca* reference genome was employed to analyze the allelic variation in the *FaTM6* locus. In this regard, a workflow has been proposed for CRISPR/Cas-mediated mutagenesis for plant species that lack genome sequence information, or feature high heterozygosity or ploidy levels^140^. This workflow could be also applicable for many horticultural crops.

### High-throughput de novo discovery of CREs in their native context

Currently, experimental validation of predicted CREs largely rely on in vitro techniques that are low accuracy and slow throughput. In recent years, new applications, such as DNase-seq (DNase I hypersensitive sites sequencing), ATAC-seq (assay for transposase-accessible chromatin using sequencing), and CHIP-seq, have significantly increased our understanding of transcriptional regulatory elements^108^. However, these techniques only provide circumstantial evidence and cannot assess the function of CREs in their native context^108^. As a complementary approach, CRISPR/Cas-based tiling screen approach was developed in mammalian cells to pinpoint functional CREs in their endogenous context^141^. The strategy is to densely tile gRNAs across a *cis*-regulatory region to map functional regulatory elements^142–146^. Although the CRISPR/Cas-based tiling screen approach has not been applied for pinpointing CREs at a large scale in plants, its feasibility was demonstrated in tomato by Rodríguez-Leal et al.^7^.

### Efficient and precise genome editing

Efficient precise genome editing is required to achieve *cis*-engineering at the nucleotide level. Base editors, including cytidine base editors (CBEs) and adenine base editors (ABEs), are efficient tools for introducing base substitutions at target sites beyond double-strand breaks^147,148^. Until now, only CBEs have been optimized and applied for gene function studies in horticultural crops, including tomato^121,149^, potato^120,121^, and watermelon^150^. Although base editors are good alternatives to HDR-mediated point mutations, it has been challenging to achieve knock-in and replacement of desired CREs in plants. Much efforts has been made to improve the efficiency of HDR through donor design and modulating repair pathways^138^. Recently, a fast and precise multiplexing genome editing method was developed in moss (*Physcomitrella patens*)^151^. Co-transformation of CRISPR/Cas9 and oligonucleotide templates introduced various mutations into the moss genome with high accuracy and efficiency. It will be interesting to apply such a fast and efficient technology in horticultural crops.

### Epigenome editing

The natural epimutations in plants illustrate the potential to further generating phenotypic variation^46^. However, only a small number of natural epialleles have been described in horticultural crops^50,52–56^. Fortunately, nuclease-dead Cas-mediated epigenome editing technologies hold great promise to expand phenotypic diversity in crops^46,47^. While some epialleles can be stably inherited over several generations, others epialleles are transient^50,152–154^. Thus, the stable transmission of editing induced epigenetic changes to the offspring remains unclear^46,155^. In addition, the expression of CRISPR components may be needed to maintain the trait in the offspring, limiting its application for crop improvement. Further development of CRISPR-based editing tools and the identification of valuable epialleles in horticultural crops will contribute to the application of epigenome editing for expanding phenotypic diversity.

## Concluding remarks

**Fig. 3:**
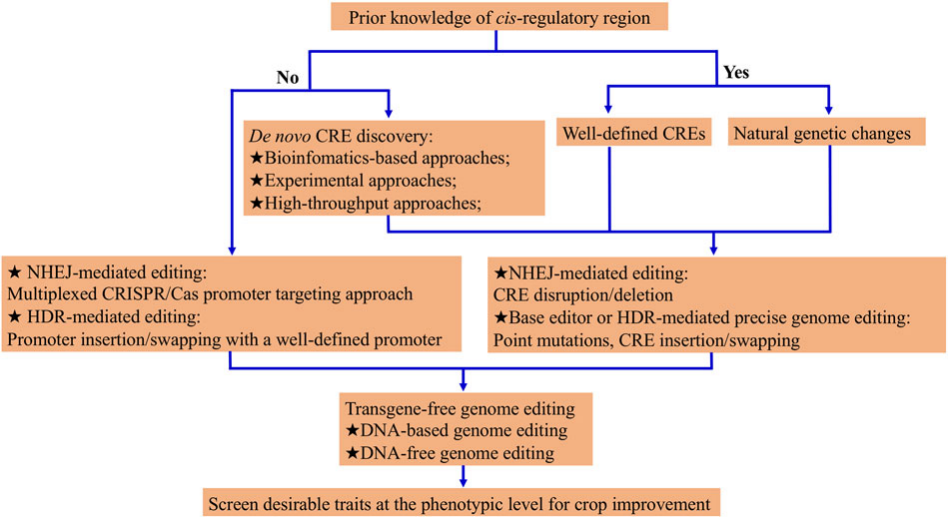
Strategies for applying CRISPR/Cas-mediated *cis*-engineering in horticultural crops.

We need to continuously improve horticultural commodities to meet the rising demand for food and ornamental production. The widespread applications of CRISPR/Cas technologies in horticultural crops open the possibility for accelerating new variety development^12–17^. Engineering *cis*-regulatory regions using CRISPR/Cas allows the creation of novel variants, resulting in quantitative variation, and thus holds great potential for creating phenotypic diversity. However, *cis*-engineering is in the beginning stages, and complex relationships between regulation of gene expression by different CREs and the resulting phenotypic changes remains to be fully elucidated^7^. Therefore, using these CRISPR/Cas techniques to screen for desirable traits at the phenotypic level rather than detecting gene expression differences is practical for crop improvement (Fig. 3). Although challenges remain, the application of CRISPR/Cas-mediated *cis*-engineering for horticultural crops improvement will further enhance breeding efforts to improve crop yield, resilience, and commercially desirable traits.
